# Beyond Cancer: Differences in Psychosocial Burden Between Patients with Chronic Non-Cancer Diagnoses Assessed Using the Integrated Palliative Outcome Scale

**DOI:** 10.3390/healthcare14131999

**Published:** 2026-07-06

**Authors:** Monika Grochowicka, Monika Pyszczorska, Grzegorz Kowalski, Małgorzata Reysner, Tomasz Reysner, Wojciech Leppert, Katarzyna Wieczorowska-Tobis

**Affiliations:** 1 Department of Palliative Medicine, Poznan University of Medical Sciences, 61-245 Poznan, Poland; monikagrochowicka@gmail.com (M.G.); monika.pyszczorska@usk.poznan.pl (M.P.); gkowalski@ump.edu.pl (G.K.); treysner@ump.edu.pl (T.R.); 2 Department of Clinical Anesthesiology and Pain Management, Poznan University of Medical Sciences, 61-701 Poznan, Poland; mreysner@ump.edu.pl; 3 Department of Palliative Medicine, Institute of Medical Sciences, Collegium Medicum, University of Zielona Góra, 65-046 Zielona Góra, Poland; wojciechleppert@wp.pl; 4 University Clinical Hospital, 60-352 Poznan, Poland

**Keywords:** IPOS-PoL, non-cancer patients, cancer patients, patient-centered care, symptom burden, psychosocial needs

## Abstract

**Background/Objectives**: The Integrated Palliative Outcome Scale (IPOS) was developed for use in patients with advanced, life-limiting conditions. This study aimed to apply the IPOS in an inpatient hospice setting in Poland, incorporating both patient- and healthcare staff-reported perspectives in cancer and non-cancer populations. **Methods**: Patients’ needs were assessed in 112 individuals (86 patients with cancer [C] and 26 with non-cancer diagnoses [nC]) using the Polish version of the IPOS. Assessments were conducted twice: within 24 h of admission (A1) and after 7 days (A2). **Results**: The mean age of the study population was 73.9 ± 11.9 years, and 63 patients (56.2%) were male. At A1, the total IPOS score reported by staff was significantly lower than that reported by patients (*p* < 0.01), primarily due to lower scores in the psychosocial domain (*p* < 0.001), while somatic domain scores were comparable. At A2, no significant changes were observed in total IPOS scores or in any domain in either patient- or staff-reported assessments. At A1, total IPOS scores did not differ significantly between C and nC groups. However, psychosocial domain scores were higher in the nC group (*p* < 0.01). Patients with non-cancer conditions reported higher levels of anxiety (*p* < 0.05), as well as greater needs related to feeling at peace (*p* < 0.01), sharing feelings (*p* < 0.05), and access to information (*p* < 0.05). **Conclusions**: Our findings underscore the multidimensional nature of suffering and highlight the need for more comprehensive recognition and assessment of psychosocial needs in palliative care patients, particularly those with non-cancer diagnoses.

## 1. Introduction

Palliative care is focused on improving the quality of life of patients with progressive, incurable, life-threatening diseases and their families [1]. It encompasses both patients with cancer and those with non-cancer diagnoses, such as heart failure, chronic obstructive pulmonary disease, or neurological disorders, who often experience a comparable or even greater burden of symptoms [2]. However, patients with non-cancer conditions more frequently present with long-standing functional impairment, which may contribute to a distinct profile of care needs [3].

The complexity of needs among patients receiving palliative care requires the use of standardized tools that enable systematic and multidimensional assessment. One of the recommended tools for assessing both patient needs and the effectiveness of palliative care is the Integrated Palliative Outcome Scale (IPOS), developed as a comprehensive instrument to evaluate physical symptoms, psychological well-being, and psychosocial and communication-related concerns [4]. A key advantage of IPOS is its ability to incorporate both the patient’s perspective and the assessment of healthcare professionals, enhancing its clinical utility. Furthermore, its availability in multiple language versions enables meaningful comparisons across different countries and healthcare settings [5].

IPOS was designed for use in patients with a wide range of advanced, life-limiting conditions. It has been applied in studies involving both cancer [6,7] and non-cancer populations [8,9]. Moreover, disease-specific adaptations have been developed to address particular symptom profiles, such as IPOS-renal for patients with advanced kidney disease [10,11] and IPOS-neuro for those with chronic neurological conditions [12,13]. Comparative studies exploring differences in needs between cancer and non-cancer populations have also been conducted [2,14]; however, such analyses have not yet been performed in Poland. This comparison is particularly relevant in the Polish palliative care setting, where palliative care services have historically been developed primarily for patients with cancer, while the growing population of patients with advanced non-cancer conditions increasingly requires access to specialist palliative care. Better understanding of the symptom burden and psychosocial needs of these patient groups may support more individualized care planning, facilitate the allocation of healthcare resources, and contribute to the development of equitable palliative care services based on patient needs rather than diagnosis alone.

The aim of this study was to apply the IPOS to assess the needs of patients receiving palliative care in an inpatient hospice setting in Poland, from both the patient’s perspective and that of the healthcare staff. This approach enables comparison of symptom burden and psychosocial needs between cancer and non-cancer patients, as well as identification of similarities and differences between these groups from dual perspectives. Such an approach has important practical implications for the standardization of patient assessment in palliative care, regardless of the underlying diagnosis. The use of IPOS across diverse patient populations supports a needs-based rather than diagnosis-based model of care, highlighting the importance of addressing both physical and psychosocial dimensions of suffering.

## 2. Materials and Methods

### 2.1. Participants

Participants were recruited from patients hospitalized in the Palliative Care Unit of the Central Clinical Hospital in Poznan, Poland. Inclusion criteria were age ≥ 18 years and the ability to maintain full logical contact, enabling comprehension of the study questions and provision of reliable responses.

All participants were informed about the study procedures and were advised of their right to withdraw from the study at any time without consequences. Written informed consent was obtained from all participants prior to enrollment.

### 2.2. Study Procedure and Instruments

The study involved a two-time-point assessment of palliative care needs conducted by both patients and formal caregivers (nursing staff). Staff assessments were completed by nurses routinely involved in the care of the participating patients. Owing to the shift-based organization of inpatient hospice care, the same nurse was not necessarily responsible for both assessments; however, all assessments were performed by experienced members of the nursing team familiar with the patients’ clinical condition.

Needs were evaluated using the Polish version of the Integrated Palliative Outcome Scale (IPOS-Pol), which has been previously validated and shown to have good psychometric properties [15].

The first assessment (Assessment 1) was performed within 24 h of admission, while the second assessment (Assessment 2) was conducted 7 days later. Recruitment continued until at least 100 patients completed both assessments, including a minimum of 25 patients with a non-cancer diagnosis. Only patients who completed both Assessment 1 and Assessment 2 were included in the final analysis. No missing data were present for the variables included in the analyses.

The IPOS is a standardized tool designed for comprehensive, multidimensional assessment of needs in patients receiving palliative care. The scale includes 10 somatic symptoms (e.g., pain, dyspnea, nausea, and fatigue) and 7 psychosocial domains (e.g., anxiety, depression, feeling at peace, concerns of the patient and their family, informational needs, and practical problems).

Each item is scored on a scale from 0 to 4, where 0 indicates no problem and 4 indicates the highest severity. Although some items (e.g., “feeling at peace” and “information”) are positively worded, all items are coded within a uniform scoring direction according to the validated IPOS scoring system. Consequently, no additional reverse scoring was applied. The total score was calculated as the sum of individual item scores, with higher total scores reflecting greater overall symptom burden. The assessment refers to the three days preceding the evaluation. The total score for somatic symptoms ranges from 0 to 40, and for psychosocial domains from 0 to 28, resulting in an overall IPOS score ranging from 0 to 68. Higher scores indicate greater symptom burden and unmet needs.

### 2.3. Statistical Analysis

The normality of data distribution was assessed using the Shapiro–Wilk test. As the majority of variables did not follow a normal distribution, results are presented as means with standard deviations (SDs) and medians with interquartile ranges (IQRs).

Paired data from the same patients obtained at two time points were compared using the Wilcoxon signed-rank test. Comparisons between patient and staff assessments, as well as between patients with cancer and non-cancer diagnoses, were performed using the Mann–Whitney U test. Agreement between patient- and staff-reported total IPOS scores was assessed using the intraclass correlation coefficient (ICC).

To account for multiple testing, Benjamini–Hochberg false discovery rate correction (FDR = 5%) was applied to all baseline comparisons between cancer and non-cancer patients. Agreement between patient- and staff-reported total IPOS scores was evaluated using the intraclass correlation coefficient (ICC; two-way mixed-effects model, absolute agreement). A multivariable linear regression model with robust HC3 standard errors was constructed to assess whether diagnosis remained independently associated with baseline psychosocial IPOS scores after adjustment for age and sex. Results are presented as regression coefficients (β) with 95% confidence intervals (95% CI). Effect sizes for Mann–Whitney U tests were reported as rank-biserial correlation coefficients (r). A *p*-value of <0.05 was considered statistically significant.

## 3. Results

The mean age of the 112 patients analyzed was 73.9 ± 11.9 years (median 73.0; IQR 67.0–84.0), and 63 (56.2%) were male. The study population included 86 patients with a cancer diagnosis and 26 with non-cancer conditions. The most common cancer diagnoses were colorectal cancer (*n* = 18; 20.9% of cancer patients) and lung cancer (*n* = 18; 20.9%). Among non-cancer patients, the most frequent diagnoses were cardiovascular diseases (*n* = 12; 46.2%) and chronic respiratory diseases (*n* = 8; 30.8%). There were no significant differences between cancer and non-cancer patients in terms of age or sex distribution. The mean age was 72.5 ± 11.7 years (median 73.0; IQR 65.5–78.0) in the cancer group and 77.4 ± 11.8 years (median 79.0; IQR 68.0–87.3) in the non-cancer group. Males constituted 57.0% (*n* = 49) of the cancer group and 53.8% (*n* = 14) of the non-cancer group.

At admission, the mean total IPOS score reported by patients was 21.3 ± 9.6 (median 21.0; IQR 13.0–27.8). Reported needs related to somatic symptoms (10.7 ± 7.1 [median 9.5; IQR 5.0–15.8] and psychosocial domains (10.6 ± 3.9 [median 10.0; IQR 8.0–13.0]) were comparable. Among somatic symptoms, low mobility was rated highest (2.4 ± 1.4; median 3.0; IQR 1.0–3.0), whereas gastrointestinal symptoms were rated lowest, including vomiting (0.3 ± 0.7; median 0; IQR 0–0) and nausea (0.5 ± 1.0; median 0; IQR 0–0), as well as shortness of breath (0.6 ± 1.1; median 0; IQR 0–1). Within the psychosocial domain, the highest scores were observed for practical problems (2.5 ± 1.7; median 4.0; IQR 1–4) and family anxiety (2.5 ± 1.5; median 3.0; IQR 1–4), while the lowest scores were reported for access to information (0.7 ± 1.2; median 0; IQR 0–1) and feeling at peace (0.8 ± 1.0; median 0; IQR 0–1).

In the staff assessment, the total IPOS score was significantly lower than that reported by patients (17.2 ± 9.1 [median 16; IQR 10.3–22] vs. 21.3 ± 9.6 [median 21.0; IQR 13.0–27.8], *p* < 0.01), primarily due to lower scores in the psychosocial domain (8.2 ± 4.6 [median 8; IQR 4–11] vs. 10.6 ± 3.9 [median 10; IQR 8–13], *p* < 0.001). However, within the psychosocial domain, staff rated only practical problems significantly lower than patients (0.7 ± 1.3 [median 0; IQR 0–1] vs. 2.5 ± 1.7 [median 4; IQR 1–4], *p* < 0.001), while other items were comparable between groups. Total scores for somatic symptoms were similar in staff and patient assessments. The only somatic symptom rated differently was mouth pain or dryness, which was assessed significantly lower by staff compared to patients (0.5 ± 1.0 [median 0; IQR 0–1] vs. 1.0 ± 1.4 [median 0; IQR 0–2], *p* < 0.01).

At the one-week follow-up (Assessment 2), no significant changes in total IPOS scores were observed in either patient or staff assessments. Similarly, total scores for somatic symptoms and the psychosocial domain (anxiety and practical problems) remained unchanged. Among somatic symptoms, patients reported a significant decrease only in constipation after one week of hospitalization (0.5 ± 1.0 [median 0; IQR 0–1] vs. 0.8 ± 1.3 [median 0; IQR 0–1.3]—*p* < 0.05). Staff assessments also indicated lower constipation scores (0.5 ± 1.0 [median 0; IQR 0–1] vs. 0.8 ± 1.3 [median 0; IQR 0–1], *p* < 0.05), but higher scores for drowsiness (1.1 ± 1.1 [median 1; IQR 0–2] vs. 0.7 ± 0.9 [median 0; IQR 0–1], *p* < 0.01).

Regarding psychosocial symptoms, both patients and staff reported reduced needs related to access to information in the second assessment (patients: 0.5 ± 1.0 [median 0; IQR 0–0] vs. 0.7 ± 1.2 [median 0; IQR 0–1], *p* < 0.05; staff: 0.3 ± 0.8 [median 0; IQR 0–0] vs. 0.5 ± 1.1 [median 1; IQR 0–2], *p* < 0.05). In contrast, family anxiety was rated significantly higher at follow-up by both patients (3.1 ± 1.4 [median 4; IQR 3–4] vs. 2.5 ± 1.5 [median 3; IQR 1–4], *p* < 0.01) and staff (3.0 ± 1.3 [median 4; IQR 2–4] vs. 2.7 ± 1.5 [median 3; IQR 2–4], *p* < 0.05).

Detailed results for all assessments are presented in Table 1.

### Comparison of IPOS Outcomes Between Patients with Cancer and Non-Cancer Diagnoses

At admission (Assessment 1), the total IPOS score did not differ significantly between patients with cancer and those with non-cancer diagnoses (20.8 ± 9.4 [median 19; IQR 13–26.5] vs. 22.6 ± 10.1 [median 22; IQR 14–31], respectively). There were also no significant differences in overall somatic symptom scores. However, non-cancer patients reported greater needs related to shortness of breath (1.0 ± 1.3 [median 0; IQR 0–2] vs. 0.5 ± 1.0 [median 0; IQR 0–1], *p* < 0.05) and lower levels of vomiting (0.0 ± 0.2 [median 0; IQR 0–0] vs. 0.4 ± 0.8 [median 0; IQR 0–2], *p* < 0.05).

In contrast, the total score for the psychosocial domain was significantly higher in non-cancer patients compared to those with cancer (12.1 ± 4.3 [median 13; IQR 8–15] vs. 10.0 ± 3.6 [median 9; IQR 8–12], *p* < 0.01). Non-cancer patients reported higher levels of patient anxiety (2.1 ± 1.3 [median 2; IQR 1–3] vs. 1.3 ± 1.3 [median 0; IQR 0–2], *p* < 0.05), as well as greater needs related to feeling at peace (1.3 ± 1.2 [median 1; IQR 0–3] vs. 0.6 ± 0.9 [median 0; IQR 0–1, *p* < 0.01), sharing feelings (1.7 ± 1.5 [median 2; IQR 0–3] vs. 1.0 ± 1.3 [median 0; IQR 0–2], *p* < 0.05), and access to information (1.1 ± 1.4 [median 1; IQR 0–2] vs. 0.6 ± 1.1 [median 0; IQR 0–0.5], *p* < 0.05). The only parameter rated lower by non-cancer patients was practical problems, both at the first assessment (2.0 ± 1.7 [median 2; IQR 0–4] vs. 2.7 ± 1.7 [median 4; IQR 1–4], *p* < 0.05) and at the second assessment (2.0 ± 1.6 [median 2; IQR 0–4] vs. 2.9 ± 1.8 [median 4; IQR 0–4], *p* < 0.05). The differences observed at baseline in somatic symptoms were no longer present at the second assessment (Table 2). The magnitude of significant between-group differences was generally small to moderate, with the largest effect observed for the psychosocial IPOS total score (r = 0.37) (Supplementary Table S2).

To evaluate whether the observed differences were independent of demographic characteristics, a multivariable linear regression analysis was performed with psychosocial IPOS score at baseline as the dependent variable and diagnosis, age, and sex as independent variables. Non-cancer diagnosis remained independently associated with higher psychosocial burden after adjustment for age and sex (β = 2.54, 95% CI 0.76–4.32, *p* = 0.005) (Table 3).

To address multiple testing, the Benjamini–Hochberg false discovery rate correction was applied to all baseline comparisons. After correction, significant differences between cancer and non-cancer patients remained for shortness of breath (q = 0.042), feeling at peace (q = 0.042), and the psychosocial IPOS total score (q = 0.042), whereas the remaining comparisons were no longer statistically significant (Supplementary Table S1). Effect sizes for significant between-group comparisons are presented in Supplementary Table S2.

To assess the agreement between patient- and staff-reported IPOS scores, an intraclass correlation coefficient (ICC) analysis was performed. For the first assessment, agreement between patient and staff ratings was moderate to good (ICC = 0.72). For the second assessment, agreement remained moderate (ICC = 0.61). Despite this level of agreement, healthcare professionals consistently reported lower total IPOS scores than patients, suggesting an underestimation of symptom burden, particularly in psychosocial domains. Detailed agreement analysis is presented in Supplementary Table S3.

Post hoc detectable change analysis was performed to evaluate the interpretation of longitudinal findings. With the available sample size, the study had 80% power to detect a paired change of approximately 2.43 points in the patient-reported total IPOS score. The observed mean change in patient-reported total IPOS score was small (−0.37 points) and not statistically significant. Therefore, no statistically significant change in overall symptom burden was observed during the one-week follow-up period (Supplementary Table S4).

## 4. Discussion

The present study, using the Integrated Palliative care Outcome Scale (IPOS), provides insight into the multidimensional burden of symptoms and needs among patients receiving palliative care. The findings confirm that symptom burden extends beyond physical complaints and includes substantial psychosocial components [16,17]. The overall IPOS scores reported by patients at admission indicate a moderate level of unmet needs, which is consistent with previous studies validating IPOS across different populations and settings [7,18]. Importantly, somatic and psychosocial domains were rated at comparable levels, emphasizing that non-physical suffering constitutes a significant proportion of the patient experience. It is also in agreement with a previous study [19].

Our results demonstrate that patients report higher symptom burden than healthcare professionals in the domains of dry mouth and practical concerns. The phenomenon of patient-clinician/caregiver discordance has been reported in previous studies [20,21] and is considered an important challenge in the assessment of patient-reported outcomes not only in palliative care [22]. The substantial discrepancy observed in the assessment of practical problems suggests that these concerns may remain insufficiently recognized by healthcare professionals. Notably, formal agreement analysis demonstrated moderate-to-good concordance between patient and staff assessments, suggesting that the observed discrepancies reflect systematic underestimation of selected domains rather than complete disagreement between raters. Unlike physical symptoms, practical issues such as financial difficulties, caregiving arrangements, administrative matters, or concerns related to future care planning are often not readily apparent during routine clinical encounters. Consequently, healthcare professionals may underestimate their importance, whereas patients may perceive them as a major source of distress. This finding highlights the limitations of relying solely on staff assessments and supports the routine use of patient-reported outcome measures such as IPOS. From a clinical perspective, systematic assessment of practical concerns may facilitate earlier involvement of social workers, psychologists, and other members of the multidisciplinary team [16,17], contributing to a more comprehensive and person-centred approach to palliative care.

In this context, the use of IPOS may be considered an important organizational and clinical tool, as it enables systematic identification of both physical and psychosocial needs that may otherwise remain underestimated by healthcare professionals.

The lack of significant improvement in total IPOS scores after one week of hospitalization reflects the chronic and complex nature of needs in palliative care populations. While some individual symptoms—such as constipation—improved, others, including drowsiness or family-related concerns, worsened over time. This pattern suggests that short-term inpatient palliative care may not lead to global improvement in symptom burden as suggested by other studies using IPOS [10,23]. At the same time, the repeated use of standardized multidimensional assessment tools may facilitate earlier recognition of dynamic changes in patient needs and support more individualized therapeutic interventions.

The changes observed in psychosocial domains in our study are particularly noteworthy. Improved access to information reported in the second assessment suggests that hospitalization in a palliative care unit may enhance communication and patient education through repeated interactions with the multidisciplinary team. These findings align with available evidence showing improved communication following consultations with a palliative care team [24]. An interesting finding was the increase in family anxiety during the follow-up period. This may reflect the progressive nature of advanced illness and the emotional burden experienced by relatives as they observe ongoing clinical deterioration. Family members often face uncertainty regarding prognosis, future care needs, and end-of-life decision-making, which may contribute to increasing distress over time. Although family anxiety was assessed indirectly through patient-reported perceptions, this finding underscores the importance of systematic psychosocial support not only for patients but also for their caregivers and relatives. Improved communication within the palliative care team may therefore need to be accompanied by targeted interventions aimed at supporting family members throughout the disease trajectory [17]. In addition, admission to a palliative care unit often coincides with disease progression and recognition of the advanced stage of illness, which may increase emotional distress among relatives. Moreover, ongoing discussions regarding prognosis and future care planning may raise awareness of the patient’s condition and contribute to greater concern among family members. This finding highlights the complex interplay between improved clinical communication and potential limitations in informal support during hospitalization. It also points to the potential value of family-centered communication models and digital support strategies aimed at maintaining family engagement during inpatient care [17,24].

A key finding of this study is that patients with non-cancer diagnoses reported a higher psychosocial burden compared to those with cancer. This observation highlights a potentially important gap in the provision of holistic palliative care, suggesting that the needs of non-cancer patients with chronic diseases may be under-recognized and insufficiently addressed [25]. Several mechanisms may potentially explain the higher psychosocial burden observed among patients with non-cancer diagnoses. Previous studies have suggested that prolonged disease trajectories, prognostic uncertainty, and differences in access to palliative care services may contribute to psychological distress in patients with advanced non-malignant diseases. However, these factors were not directly assessed in the present study. Therefore, the proposed explanations should be considered hypothesis-generating rather than definitive interpretations of the observed findings. Importantly, overall IPOS scores and overall somatic symptom burden did not differ significantly between cancer and non-cancer patients, and the observed differences were primarily confined to the psychosocial domain [3,26]. This unpredictability may contribute to increased psychological distress, anxiety, and a sense of insecurity among patients, thereby intensifying the psychosocial dimension of suffering [27,28]. Importantly, this association remained significant after adjustment for age and sex in multivariable analysis, suggesting that the observed difference was not solely explained by demographic characteristics. Moreover, effect size estimates indicated that the observed differences were generally small to moderate, with the strongest effect for the overall psychosocial burden. An important methodological consideration relates to the use of IPOS-Pol in patients with non-cancer diagnoses. Although IPOS was originally developed as a multidimensional outcome measure for palliative care populations regardless of diagnosis and has been widely applied internationally in both cancer and non-cancer settings, the Polish validation study was conducted primarily in patients with advanced cancer. Consequently, the psychometric properties of IPOS-Pol may differ in non-cancer populations. This limitation should be considered when interpreting the observed differences between cancer and non-cancer patients. Nevertheless, the use of IPOS in non-cancer populations is supported by previous studies conducted in patients with advanced heart failure, chronic kidney disease, and neurological disorders [8,9,10,11,12,13]. Further validation studies of IPOS-Pol in non-cancer palliative care populations are needed.

The observed differences may also reflect systemic factors. Palliative care models have historically been developed within the oncological framework [29], which may result in more structured support pathways for patients with cancer. Consequently, individuals with non-cancer conditions may have less access to comprehensive psychosocial support, leading to a higher burden of unmet needs. Importantly, some of the observed differences persisted over time, suggesting that standard inpatient care may be insufficient to address psychosocial dimensions of suffering, particularly in non-cancer populations.

However, the heterogeneity of the non-cancer group should also be taken into account. This group comprised patients with a range of advanced chronic diseases, including cardiovascular and respiratory conditions, each associated with different symptom burdens and clinical trajectories. Consequently, some of the observed differences, particularly regarding symptoms such as breathlessness, may be attributable to disease-specific characteristics rather than reflecting general differences between cancer and non-cancer populations. Therefore, these findings should be interpreted with caution and confirmed in studies involving larger, more diagnostically homogeneous patient groups.

Our study has several limitations. First, although IPOS-Pol has been formally validated in patients with advanced cancer and has been increasingly used in non-cancer palliative care populations internationally, its psychometric properties have not yet been comprehensively evaluated in Polish-speaking patients with non-cancer diagnoses. Therefore, comparisons between cancer and non-cancer patients should be interpreted with caution. Second, the relatively small and heterogeneous non-cancer cohort may limit the generalizability of the findings. Observed differences in symptoms, such as breathlessness, may reflect disease-specific characteristics rather than universal differences between cancer and non-cancer populations. In addition, important clinical variables, including performance status, disease severity, comorbidity burden, duration of illness, prior exposure to palliative care, and prognostic indicators, were not collected. Consequently, it was not possible to determine whether the observed differences were attributable to the diagnosis itself or to differences in overall clinical status. Third, the study was conducted in a single inpatient palliative care unit and included only patients who were able to maintain full logical contact and independently complete the IPOS assessment. Patients with cognitive impairment, delirium, advanced neurological deficits, or severe clinical deterioration were therefore not represented. These factors may limit the external validity of the findings and their applicability to the broader palliative care population. Finally, the longitudinal findings should be interpreted cautiously. The follow-up period was limited to seven days and may have been insufficient to detect clinically meaningful changes in symptom burden. Moreover, the study was powered to detect changes of approximately 2.4 points in the patient-reported total IPOS score; therefore, smaller longitudinal changes may have remained undetected. Several potential explanatory factors discussed in this manuscript, including disease trajectory, prognostic uncertainty, and access to psychosocial support, were not directly measured and should therefore be considered exploratory and hypothesis-generating.

## 5. Conclusions

Our findings suggest that patients with non-cancer diagnoses may experience a greater psychosocial burden than patients with cancer; however, this observation should be interpreted cautiously, given the heterogeneity and limited size of the non-cancer cohort. This highlights the importance of systematic assessment using validated tools such as IPOS. Furthermore, implementing structured patient-reported outcome measures may support more equitable and personalized palliative care by enabling earlier identification of vulnerable patient groups and tailoring interventions to their specific needs.

Taken together, our findings underline the need for more comprehensive approaches to palliative care that integrate regular multidimensional assessment, strengthen psychosocial support, and ensure equitable access to holistic care for both cancer and non-cancer populations.

## Figures and Tables

**Table 1:** The results of the IPOS-Pol assessed at admission (Assessment 1) and one week later (Assessment 2), from both the patient and staff perspectives (*n* = 112).

	Assessment 1		Assessment 2	
Somatic symptoms	Patient	Staff	Patient	Staff
Pain	1.3 ± 1.3 (1; 0–2)	1.1 ± 1.3 (1; 0–2)	1.2 ± 1.3 (1; 0–2)	1.1 ± 1.2 (1; 0–2)
Shortness of breath	0.6 ± 1.1 (0; 0–1)	0.6 ± 1.0 (0; 0–1)	0.6 ± 1.1 (0; 0–1)	0.5 ± 0.8 (0; 0–1)
Weakness or lack of energy	1.7 ± 1.3 (2; 1–3)	1.5 ± 1.3 (1; 0–3)	1.7 ± 1.5 (1; 0–2)	1.6 ± 1.4 (1; 0–3)
Nausea	0.5 ± 1.0 (0; 0–0)	0.4 ± 0.9 (0; 0–0)	0.3 ± 0.8 (0; 0–0)	0.3 ± 0.7 (0; 0–0)
Vomiting	0.3 ± 0.7 (0; 0–0)	0.2 ± 0.8 (0; 0–0)	0.2 ± 0.5 (0; 0–0)	0.1 ± 0.4 (0; 0–0)
Poor appetite	1.1 ± 1.3 (1; 0–2)	1.0 ± 1.2 (1; 0–2)	1.1 ± 1.3 (1; 0–2)	1.0 ± 1.3 (0; 0–1)
Constipation	0.8 ± 1.3 (0;0–1.3)	0.8 ± 1.3 (0;0–1)	0.5 ± 1.0 # (0; 0–1)	0.5 ± 1.0 # (0; 0–1)
Mouth pain or dryness	1.0 ± 1.4 (0; 0–2)	0.5 ± 1.0 ** (0; 0–1)	0.7 ± 1.1 (0; 0–1)	0.5 ± 0.9 (0; 0–1)
Drowsiness	1.0 ± 1.2 (1; 0–2)	0.7 ± 0.9 (0; 0–1)	1.2 ± 1.2 (1; 0–2)	1.1 ± 1.1 ## (1; 0–2)
Low mobility	2.4 ± 1.4 (3; 1–3)	2.2 ± 1.4 (2; 1–3)	2.3 ± 1.3 (2; 1–3)	2.1 ± 1.4 (2; 1–3)
Overall	10.7 ± 7.1 (9.5; 5–15.8)	9.0 ± 6.1 (8; 4.3–12)	9.8 ± 6.3 (8; 5–14)	8.6 ± 5.5 (7; 5–12.8)
Psychosocial symptoms	Patient	Staff	Patient	Staff
Patient anxiety (Did you feel anxious due to illness or treatment?)	1.5 ± 1.3 (2; 0–3)	1.3 ± 1.3 (1; 0–2)	1.7 ± 1.3 (2; 0–3)	1.4 ± 1.2 (1; 0–2)
Family anxiety (Did anyone in your family or friends worry about you?)	2.5 ± 1.5 (3; 1–4)	2.7 ± 1.5 (3; 2–4)	3.1 ± 1.4 ## (4; 3–4)	3.0 ± 1.3 # (4; 2–4)
Depression (Did you feel depressed?)	1.3 ± 1.2 (1; 0–2)	1.2 ± 1.3 (1; 0–2)	1.4 ± 1.2 (1; 0–2)	1.4 ± 1.2 (1; 0–2)
Feeling at peace (Did you feel calm?)	0.8 ± 1.0 (0; 0–1)	0.7 ± 0.9 (0; 0–1)	0.8 ± 1.0 (0; 0–1)	0.7 ± 0.9 (0; 0–1)
Sharing feelings (Were you able to share with your family/friends how you feel to the extent you expected?)	1.2 ± 1.4 (0; 0–2)	1.1 ± 1.3 (1; 0–2)	1.0 ± 1.3 (0; 0–2)	1.0 ± 1.3 (0; 0–2)
Information (Were you informed to the extent you expected?)	0.7 ± 1.2 (0; 0–1)	0.5 ± 1.1 (1; 0–2)	0.5 ± 1.0 # (0; 0–0)	0.3 ± 0.8 # (0; 0–0)
Practical matters (Were any practical problems addressed?)	2.5 ± 1.7 (4; 1–4)	0.7 ± 1.3 *** (0; 0–1)	2.7 ± 1.8 (4; 1–4)	0.7 ± 1.3 *** (0; 0–1)
Total anxiety-practical problems	10.6 ± 3.9 (10; 8–13)	8.2 ± 4.6 *** (8; 4–11)	11.1 ± 3.8 (11; 9–14)	8.6 ± 3.8 *** (8.5; 5.3–11)
Total Score	21.3 ± 9.6 (21; 13–27.8)	17.2 ± 9.1 ** (16; 10.3–22)	20.9 ± 8.6 (19; 15.3–26.8)	17.2 ± 8.2 *** (16; 12–20.8)

Statistical analysis: patient vs. staff: **—*p* < 0.01; ***—*p* < 0.001; assessment 1 vs. assessment 2: #—*p* < 0.05; ##—*p* < 0.01).

**Table 2:** The results of the IPOS assessed at admission (Assessment 1) and one week later (Assessment 2), from both the patients with cancer (*n* = 86) and non-cancer diagnoses (*n* = 26).

	Assessment 1		Assessment 2	
Somatic symptoms	Patients with cancer diagnoses	Patients with non-cancer diagnoses	Patients with cancer diagnoses	Patients with non-cancer diagnoses
Pain	1.2 ± 1.3 (1; 0–2)	1.3 ± 1.3 (1; 0–3)	1.2 ± 1.3 (1; 0–2)	1.2 ± 1.2 (1; 0–2)
Shortness of breath	0.5 ± 1.0 (0; 0–1)	1.0 ± 1.3 ** (0; 0–2)	0.4 ± 1.0 (0; 0–0)	0.9 ± 1.3 (0; 0–1)
Weakness or lack of energy	1.7 ± 1.2 (2; 1–3)	1.9 ± 1.4 (2; 1–3)	1.7 ± 1.4 (2; 0–3)	1.6 ± 1.5 (1; 0–3)
Nausea	0.6 ± 1.1 (0; 0–1)	0.3 ± 0.8 (0; 0–0)	0.4 ± 0.9 (0; 0–0)	0.2 ± 0.4 (0; 0–0)
Vomiting	0.4 ± 0.8 (0; 0–0)	0.0 ± 0.2 * (0; 0–0)	0.2 ± 0.6 (0; 0–0)	0.1 ± 0.4 (0; 0–0)
Poor appetite	1.2 ± 1.3 (0; 0–2)	0.7 ± 1.0 (0; 0–1)	1.2 ± 1.4 (1; 0–4)	0.8 ± 1.0 (0; 0–2)
Constipation	0.9 ± 1.4 (0; 0–2)	0.6 ± 1.0 (0; 0–1)	0.5 ± 1.1 ## (0; 0–0)	0.5 ± 0.9 (0; 0–1)
Mouth pain or dryness	1.0 ± 1.4 (0; 0–2)	1.0 ± 1.4 (0; 0–2)	0.6 ± 1.1 (0; 0–1)	0.9 ± 1.2 (0; 0–2)
Drowsiness	1.0 ± 1.1 (1; 0–2)	1.2 ± 1.4 (1; 0–2)	1.2 ± 1.1 (1; 0–2)	1.3 ± 1.4 (1; 0–2)
Low mobility	2.4 ± 1.4 (3; 1–3.5)	2.4 ± 1.3 (3; 1–3)	2.3 ± 1.3 (3; 1–3)	2.2 ± 1.4 (2; 1–3)
Overall	10.8 ± 7.3 (9; 5–16)	10.5 ± 6.8 (11; 4–15)	9.8 ± 6.4 (8; 5–14)	9.7 ± 6.1 (9; 4–15)
Psychosocial symptoms	Patients with cancer diagnoses	Patients with non-cancer diagnoses	Patients with cancer diagnoses	Patients with non-cancer diagnoses
Patient anxiety (Did you feel anxious due to illness or treatment?)	1.3 ± 1.3 (1; 0–2)	2.1 ± 1.3 * (2; 1–3)	1.7 ± 1.3 (1; 0–2,5)	2.0 ± 1.3 (2; 0–3)
Family anxiety (Did anyone in your family or friends worry about you?)	2.6 ± 1.6 (3; 1–4)	2.3 ± 1.4 (2; 1–4)	3.1 ± 1.4 ## (4; 3–4)	3.0 ± 1.4 (4; 2–4)
Depression (Did you feel depressed?)	1.2 ± 1.2 (1; 0–2)	1.6 ± 1.1 (2; 1–2)	1.4 ± 1.2 (1; 0–2)	1.7 ± 1.1 (2; 1–2)
Feeling at peace (Did you feel calm?)	0.6 ± 0.9 (0; 0–1)	1.3 ± 1.2 ** (1; 0–3)	0.6 ± 0.9 (0; 0–1)	1.3 ± 1.2 * (0; 0–2)
Sharing feelings (Were you able to share with your family/friends how you feel to the extent you expected?)	1.0 ± 1.3 (0; 0–2)	1.7 ± 1.5 * (2; 0–3)	1.0 ± 1.3 (0; 0–2)	1.4 ± 1.3 (1; 0–3)
Information (Were you informed to the extent you expected?)	0.6 ± 1.1 (0; 0–0.5)	1.1 ± 1.4 * (1; 0–2)	0.4 ± 1.0 # (0; 0–0)	0.7 ± 1.1 * (0; 0–1)
Practical matters (Were any practical problems addressed?)	2.7 ± 1.7 (4; 1–4)	2.0 ± 1.7 * (2; 0–4)	2.9 ± 1.8 (4; 1–4)	2.0 ± 1.6 * (2; 0–4)
Total anxiety-practical problems	10.0 ± 3.6 (9; 8–12)	12.1 ± 4.3 ** (13; 8–15)	10.8 ± 3.3 (11; 9–13)	12.0 ± 4.9 *** (13; 8–15)
Total Score	20.8 ± 9.4 (19; 13–26.5)	22.6 ± 10.1 (22; 14–31)	20.8 ± 8.3 (19; 15.5–25.5)	21.6 ± 9.7 (20; 15–29)

Statistical analysis: patients with cancer diagnoses vs. patients with non-cancer diagnoses: *—*p* < 0.05; **—*p* < 0.01; ***—*p* < 0.001 assessment 1 vs. assessment 2: #—*p* < 0.05; ##—*p* < 0.01).

**Table 3:** Multivariable linear regression analysis of factors associated with baseline psychosocial IPOS score (A1).

Predictor	β Coefficient	95% CI	*p*-Value
Non-cancer diagnosis	2.54	0.76 to 4.32	0.005
Age (years)	−0.03	−0.09 to 0.03	0.387
Male sex	−0.86	−2.33 to 0.60	0.249

Dependent variable: baseline psychosocial IPOS score (A1 psychosocial total). Robust HC3 standard errors were used.

## Data Availability

The raw data supporting the conclusions of this article will be made available by the corresponding author on request.

## Supplementary Materials

The following supporting information can be downloaded at: https://www.mdpi.com/article/10.3390/healthcare14131999/s1, Supplementary Table S1. Benjamini–Hochberg correction for baseline (A1) comparisons between cancer and non-cancer patients. Supplementary Table S2. Effect sizes for significant differences between cancer and non-cancer patients. Supplementary Table S3. Agreement between patient and staff IPOS assessments. Supplementary Table S4. Post hoc detectable change analysis for longitudinal IPOS outcomes.

## Abbreviations

The following abbreviations are used in this manuscript:

IPOS	Integrated Palliative Outcome Scale
IPOS-Pol	Polish version of the Integrated Palliative Outcome Scale
IQR	Interquartile ranges
