# Supplementary material for: Beyond Cancer: Differences in Psychosocial Burden Between Patients with Chronic Non-Cancer Diagnoses Assessed Using the Integrated Palliative Outcome Scale
Source: Healthcare (Basel). 2026 Jul 6;14(13):1999. doi: 10.3390/healthcare14131999 (PMC13362464; doi:10.3390/healthcare14131999)
Supplement: Supplementary file 1 [file healthcare-14-01999-s001.zip › healthcare-4339547-supplementary.pdf]

Supplementary tables

Supplementary Table S1. Benjamini–Hochberg correction for baseline (A1) comparisons between cancer and non-cancer patients.

Outcome	Raw p-value	BH-adjusted q-value (within A1 outcomes)	Significant after correction
Pain	0.592	0.757	No
Shortness of breath	0.006	**0.042**	**Yes**
Weakness/lack of energy	0.606	0.757	No
Nausea	0.266	0.409	No
Vomiting	0.032	0.105	No
Poor appetite	0.162	0.323	No
Constipation	0.802	0.891	No
Mouth pain/dryness	0.957	0.997	No
Drowsiness	0.214	0.389	No
Low mobility	0.576	0.757	No
Somatic total	0.751	0.883	No
Patient anxiety	0.012	0.060	No
Family anxiety	0.997	0.997	No
Depression	0.075	0.167	No
Feeling at peace	0.004	**0.042**	**Yes**
Sharing feelings	0.043	0.108	No
Information	0.017	0.068	No
Practical matters	0.042	0.108	No
Psychosocial total	0.004	**0.042**	**Yes**
Total IPOS	0.237	0.395	No

Statistical analysis: patients with cancer diagnoses vs. patients with non-cancer diagnoses: * p<0.05; ** p<0.01. Assessment 1 vs. Assessment 2: # p<0.05; ## p<0.01. After Benjamini–Hochberg correction for multiple testing, significant baseline between-group differences remained for shortness of breath (q = 0.042), feeling at peace (q = 0.042), and psychosocial IPOS total score (q = 0.042) (Supplementary Table S1).

Supplementary Table S2. Effect sizes for significant differences between cancer and non-cancer patients.

Variable	Cancer patients Mean ± SD (Median, IQR)	Non-cancer patients Mean ± SD (Median, IQR)	Effect size (r)
**Assessment 1**			
Shortness of breath	0.5 ± 1.0 (0; 0–1)	1.0 ± 1.3 (0; 0–2)	0.30
Vomiting	0.4 ± 0.8 (0; 0–0)	0.0 ± 0.2 (0; 0–0)	-0.19
Patient anxiety	1.3 ± 1.3 (1; 0–2)	2.1 ± 1.3 (2; 1–3)	0.31
Feeling at peace	0.6 ± 0.9 (0; 0–1)	1.3 ± 1.2 (1; 0–3)	0.34
Sharing feelings	1.0 ± 1.3 (0; 0–2)	1.7 ± 1.5 (2; 0–3)	0.24
Information	0.6 ± 1.1 (0; 0–0.5)	1.1 ± 1.4 (1; 0–2)	0.26
Practical matters	2.7 ± 1.7 (4; 1–4)	2.0 ± 1.7 (2; 0–4)	-0.24
Psychosocial IPOS total score	10.0 ± 3.6 (9; 8–12)	12.1 ± 4.3 (13; 8–15)	0.37
**Assessment 2**			
Feeling at peace	0.6 ± 0.9 (0; 0–1)	1.3 ± 1.2 (0; 0–2)	0.30
Information	0.4 ± 1.0 (0; 0–0)	0.7 ± 1.1 (0; 0–1)	0.25
Practical matters	2.9 ± 1.8 (4; 1–4)	2.0 ± 1.6 (2; 0–4)	-0.31

Effect sizes are reported as rank-biserial correlation coefficients (r) derived from Mann–Whitney U tests. Positive values indicate higher scores among patients with non-cancer diagnoses, whereas negative values indicate higher scores among patients with cancer diagnoses.

Supplementary Table S3. Agreement between patient and staff IPOS assessments.

Outcome	Assessment 1	Assessment 2
ICC	0.72	0.61
Interpretation	Moderate-good	Moderate
Mean patient IPOS	21.3	20.9
Mean staff IPOS	17.2	17.2

Supplementary Table S4. Post hoc detectable change analysis for longitudinal IPOS outcomes.

Outcome	N	A1 mean ± SD	A2 mean ± SD	Mean change	Wilcoxon p-value	Minimum detectable change with 80% power
Patient total IPOS	112	21.28 ± 9.58	20.91 ± 8.56	-0.37	0.869	2.43
Patient somatic domain	112	10.67 ± 7.12	9.78 ± 6.33	-0.89	0.227	1.83
Patient psychosocial domain	112	10.61 ± 3.90	11.13 ± 3.83	+0.53	0.118	1.03
Staff total IPOS	110	17.26 ± 9.14	17.10 ± 8.15	-0.16	0.937	2.21
Staff somatic domain	111	8.99 ± 6.14	8.61 ± 5.57	-0.38	0.570	1.61
Staff psychosocial domain	109	8.23 ± 4.66	8.56 ± 3.83	+0.33	0.625	1.06
